# Lightweight Ghost Enhanced Feature Attention Network: An Efficient Intelligent Fault Diagnosis Method under Various Working Conditions

**DOI:** 10.3390/s24113691

**Published:** 2024-06-06

**Authors:** Huaihao Dong, Kai Zheng, Siguo Wen, Zheng Zhang, Yuyang Li, Bobin Zhu

**Affiliations:** 1 School of Advanced Manufacturing Engineering, Chongqing University of Posts and Telecommunications, Chongqing 400065, China; 2021214581@stu.cqupt.edu.cn (H.D.); 2020213923@stu.cqupt.edu.cn (S.W.); 2022214486@stu.cqupt.edu.cn (Y.L.); 2 Chengdu Tianyou Tangyuan Engineering Testing Consulting Co., Ltd., Chengdu 610056, China; swjtuzz@163.com; 3 School of Mechanical Engineering, Inner Mongolia University of Science and Technology, Baotou 014010, China; 2164304727@stu.imust.edu.cn

**Keywords:** fault diagnosis, complex working conditions, lightweight, rolling bearing, deep learning, MobileVit, GEFA-Net

## Abstract

Recent advancements in applications of deep neural network for bearing fault diagnosis under variable operating conditions have shown promising outcomes. However, these approaches are limited in practical applications due to the complexity of neural networks, which require substantial computational resources, thereby hindering the advancement of automated diagnostic tools. To overcome these limitations, this study introduces a new fault diagnosis framework that incorporates a tri-channel preprocessing module for multidimensional feature extraction, coupled with an innovative diagnostic architecture known as the Lightweight Ghost Enhanced Feature Attention Network (GEFA-Net). This system is adept at identifying rolling bearing faults across diverse operational conditions. The FFE module utilizes advanced techniques such as Fast Fourier Transform (FFT), Frequency Weighted Energy Operator (FWEO), and Signal Envelope Analysis to refine signal processing in complex environments. Concurrently, GEFA-Net employs the Ghost Module and the Efficient Pyramid Squared Attention (EPSA) mechanism, which enhances feature representation and generates additional feature maps through linear operations, thereby reducing computational demands. This methodology not only significantly lowers the parameter count of the model, promoting a more streamlined architectural framework, but also improves diagnostic speed. Additionally, the model exhibits enhanced diagnostic accuracy in challenging conditions through the effective synthesis of local and global data contexts. Experimental validation using datasets from the University of Ottawa and our dataset confirms that the framework not only achieves superior diagnostic accuracy but also reduces computational complexity and accelerates detection processes. These findings highlight the robustness of the framework for bearing fault diagnosis under varying operational conditions, showcasing its broad applicational potential in industrial settings. The parameter count was decreased by 63.74% compared to MobileVit, and the recorded diagnostic accuracies were 98.53% and 99.98% for the respective datasets.

## 1. Introduction

Rotating machinery is an important part of modern industry [1,2,3]. Rolling bearings, which are commonly used components in rotating machinery, have a high failure rate [4,5]. The timely diagnosis of rolling bearing faults is significant for equipment safety and stability [6,7]. However, early fault features are weak and easily obscured by noise [8,9]. Complex working conditions, such as speed fluctuation and load, add challenges to fault diagnosis [10,11].

Fault diagnosis methodologies can be broadly classified into two categories: traditional signal processing methods and machine-learning-based techniques [12,13]. Traditional methods, including Empirical Mode Decomposition (EMD), Singular Value Decomposition (SVD), Wavelet Transform (WT), Wavelet Packet Transform (WPT), Empirical Wavelet Transform (EWT), and Variational Mode Decomposition (VMD), have established a foundation for initial fault analysis. However, these methods often fall short under fluctuating operational conditions due to their inherent limitations in handling complex signal characteristics, which requires expert interpretation that is frequently challenging [14,15,16,17,18,19,20]. Conversely, machine learning approaches such as Principal Component Analysis (PCA), Support Vector Machines (SVMs), and Artificial Neural Networks (ANNs) [21,22] show promise due to their adaptability and learning capabilities, though they remain sensitive to parameter settings, which may result in the loss of critical diagnostic information under nonlinear and complex fault conditions [23,24,25,26].

Intelligent fault diagnosis techniques have become increasingly crucial in the field of fault detection and diagnosis, attracting significant attention in recent years [27]. Advances in ANNs have led to the development of new models that significantly enhance diagnostic accuracy for bearing faults. Notable innovations include the use of deep transfer learning models that combine techniques such as Wavelet Packet Transform (WPT) and Residual Networks (ResNets) to enhance diagnostic precision [28]. Further, some approaches have leveraged transfer learning and time–frequency image transformations in conjunction with deep learning frameworks, thereby bolstering the robustness of fault-detection mechanisms [29]. Techniques that utilize Gramian Angular Field (GAFs) for feature mapping, coupled with network optimization strategies such as Efficient Channel Attention (ECA), have been developed, providing efficacious solutions for fault diagnosis [30]. Additionally, the amalgamation of multi-signal fusion and Markov Transfer Fields (MTFs) with ResNet has been advocated to facilitate diagnoses under variable conditions [31]. Zhang et al. [32] introduced an ensemble Swin-LE Transformer model incorporating residuals to augment fault feature extraction capabilities. Concurrently, Tang et al. [33] devised an integrated Vision Transformer (ViT) model that combines WT with a soft voting methodology. Yang et al. [34] developed a bearing fault diagnosis approach utilizing a Signal Transformer Neural Network (SiT) and Amplitude Modulation (AM). Furthermore, Ding et al. [35] proposed a new Time–Frequency Transformer (TFT) model, inspired by the substantial success of the conventional Transformer in sequence processing. Pei et al. [36] innovated a Transformer Convolution Network (TCN) grounded in transfer learning principles.

Although existing methods for fault diagnosis have demonstrated commendable performance, they generally overlook the significant dependency of deep learning frameworks on substantial hardware rigs and intensive computational processes. In scenarios characterized by limited computational resources and storage capacities, the deployment of these methodologies is notably challenging [37,38]. Indeed, the restricted computational and storage resources severely constrain the application and widespread adoption of intelligent fault diagnosis systems [39,40,41]. Consequently, there is a growing interest in exploring lightweight Convolutional Neural Network (CNN) architectures such as MobileNet [42,43] and the MobileVit [44,45,46] model, which amalgamates CNN and Transformer architectures, tailored for mobile or embedded devices. Despite their application in diagnosing bearing faults under complex operational conditions, these models continue to grapple with the challenge of inadequate computational and storage capacities [47]. To address these limitations, we have developed a lightweight fault diagnosis model that not only substantially reduces the parameter count but also enhances diagnostic accuracy and operational speed, outperforming MobileNet and MobileVit in effectiveness.

This work introduces a new framework to tackle the challenges posed by the feature diversity of the same fault category under different operational conditions. We propose a three-channel (FFT, FWEO, Envelopment) feature extraction preprocessing module (FFE) that captures and integrates features from the time, frequency, and time–frequency domains of vibration signals, utilizing techniques like the GAF for effective feature synthesis [30]. This integration not only enhances the differentiation of fault categories but also optimizes the feature extraction process, thus significantly reducing the computational load. The network leverages advanced architectures, such as MobileVit and Context Broadcasting (CB) modules, and incorporates strategic enhancements like GhostNet and EPSANet. These innovations not only elevate diagnostic accuracy but also significantly reduce the model’s parameter count, thereby enhancing the efficiency of the diagnostic process and accelerating detection speeds [48,49,50]. The main contributions of this paper are as follows:

- Developing a three-channel feature extraction module, FFE, that effectively amalgamates the time domain, the frequency domain, and the time–frequency domain features for enhanced fault identification under diverse conditions.
- Enhancing the MobileVit architecture by integrating advanced modules such as GhostNet, EPSANet, CB, and EPSA, substantially boosting both the efficiency of parameter utilization and the accuracy of fault diagnosis.
- Introducing a new diagnostic framework that addresses the significant variability in fault features across different operational conditions. Demonstrating through empirical studies that our proposed method not only improves recognition accuracy but also significantly reduces computational demands, outperforming existing models.

## 2. Preliminary

### 2.1. Frequency Weighted Energy Operator

In this study, the FWEO is introduced as a new signal processing method to improve the detection of rolling bearing fault characteristics under variable operating conditions [51,52]. FWEO represents a highly efficient signal processing technique, specifically designed to enhance fault detection by incorporating the frequency characteristics of the signal. Moreover, this method is especially suitable for analyzing complex bearing fault vibration signals in noisy environments. For a continuous-time signal *x(t)*, its FWEO is formally defined as

(1) $$\Gamma x\left(t\right)={\left(\frac{d}{dt}x\left(t\right)\right)}^{2}+{\left(\frac{d}{dt}H\left[x\left(t\right)\right]\right)}^{2},$$

where H[x(t)] is the Hilbert transform of x(t).

### 2.2. Materials and Methods: Evolved Sign Momentum Optimizer

The Evolved Sign Momentum optimizer (Lion optimizer) is a simple yet effective optimization algorithm for training deep neural networks [53]. To improve the efficiency of model training, this paper substitutes the original AdamW optimizer with the Lion optimizer. The Lion optimizer distinguishes itself from typical adaptive optimizers by exclusively tracking momentum and using sign operations to compute update magnitudes. This approach ensures lower memory overhead and uniform update magnitudes across all dimensions, significantly diverging from the mechanisms employed by adaptive optimizers. This design not only reduces memory requirements but also introduces a form of regularization, enhancing the model’s generalization capabilities. The update mechanism of the Lion optimizer is as follows:

(2) $$m_{t}=\beta \cdot m_{t-1}+\left(1-\beta \right)\cdot g_{t},$$

where $m_{t}$ represents the momentum of the current step. $m_{t-1}$ denotes the momentum of the previous step, while $g_{t}$ symbolizes the current gradient. The coefficient β is identified as the momentum decay factor. To compute the update direction, the Lion optimizer employs a symbolic operation that does not depend on the gradient’s magnitude. Consequently, the update step of the Lion optimizer is consistent across all parameters. The symbolic function is delineated as follows:

(3) $$\mathrm{sign}\left(x\right)=\left\{\begin{array}{c} -1,\;\mathrm{if}\;x<0,\\ 0,\;\mathrm{if}\;x=0,\\ 1,\;\mathrm{if}\;x>0. \end{array}\right.$$

Within the Lion optimizer framework, the update step is predicated upon the sign of the momentum rather than its magnitude:

(4) $$\Delta wt=-\eta \cdot \mathrm{sign}\left(m_{t}\right),$$

where Δwt is the update of the parameters at time step *t* and η is the learning rate. Relative to the AdamW optimizer, the Lion optimizer demonstrates enhanced training speed and greater efficiency in handling large-scale data and models. Chen [53] suggested that the Lion optimizer significantly enhances the accuracy of the ViT model on ImageNet for image classification tasks, achieving up to a fivefold reduction in pre-training computational costs.

Consequently, the Lion offers several advantages over the Adam, including improved memory efficiency, uniform update magnitudes, better performance, robustness to hyperparameter choices, and scalability with batch size. These benefits are particularly crucial in scenarios involving high-dimensional data and complex model architectures, where traditional optimizers may struggle with convergence and stability issues. The Lion optimizer’s design ensures lower memory overhead, facilitating more efficient use of computational resources and enhancing overall training speed. Additionally, its robustness to hyperparameter variations reduces the need for extensive tuning, making it a more practical choice for diverse applications. Based on these advantages, we replace the original Adam optimizer with the Lion optimizer in this study, aiming to leverage its superior performance characteristics and achieve more reliable and efficient model training outcomes.

## 3. Methodology

### 3.1. Overview

**Figure 1:**
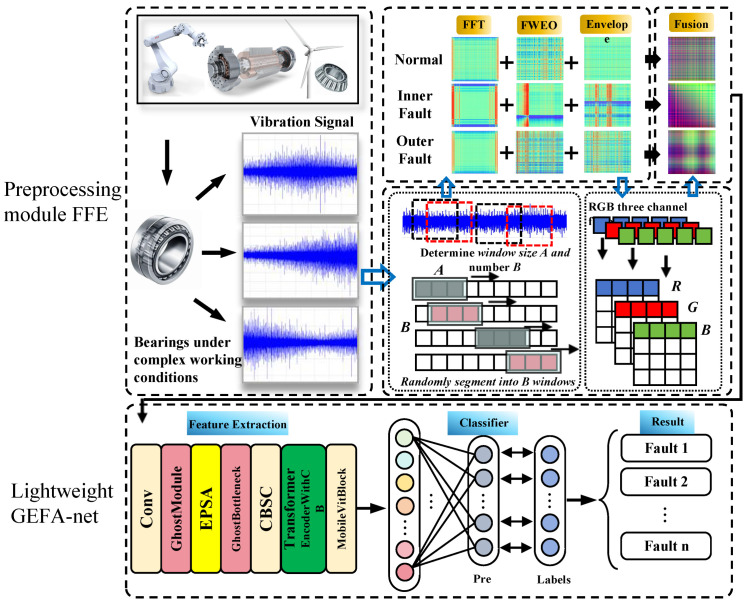
Adaptive bearing fault diagnosis framework with the preprocessing module FFE and Lightweight GEFA-Net.

In this section, a new fault diagnosis framework, designated as the Adaptive Bearing Fault Diagnosis Framework with FFE Preprocessing and Lightweight Ghost Enhanced Feature Attention Network, is introduced. As depicted in Figure 1, the process begins with the application of the FFE to extract essential features of the fault signal, capturing its time–frequency characteristics, periodicity, transient attributes, and nonlinear properties. These feature sequences are then converted into 2D image representations using the GAF. Finally, these image representations are transformed into three-channel RGB images through a channel fusion technique. Ultimately, the GEFA-Net is employed for the training, validation, and testing phases. The experimental results demonstrate that this method not only enhances diagnostic accuracy but also significantly reduces the number of model parameters and computational complexity. Table 1 presents the steps involved in the fault diagnosis framework. FFE and GEFA-Net are described in detail in the subsequent sections.

### 3.2. Preprocessing Module FFE

This section elaborates on the proposed preprocessing module FFE. Under complex operating conditions, the signal characteristics of the same rolling bearing fault can exhibit considerable variability. The FFE employs a multidimensional feature extraction strategy that integrates the time domain, frequency domain, and time–frequency domain signal characteristics to achieve deep vibration signal feature extraction. Specifically, the first channel employs the FFT to analyze the periodicity and frequency distribution of the vibration signals, thereby capturing the fundamental structure of the fault signals. The second channel utilizes the FWEO to accentuate the frequency characteristics of the signal and its energy distribution, thereby effectively isolating the key fault features. The third channel employs the signal envelopment technique to scrutinize the transient and nonlinear characteristics of the vibration signals, aiming for a comprehensive capture of the subtle changes in the fault signals. Additionally, this module introduces an innovative feature fusion strategy designed to mitigate the interference of different working conditions on fault signal diagnosis. By fusing diverse features extracted from the three channels, a comprehensive feature dataset is established. The fault characterization of this dataset is more pronounced, which yields a rich and precise information base for the subsequent machine learning model, thereby significantly enhancing the efficiency and accuracy of the model in diagnosing bearing faults under complex working conditions.

**Figure 2:**
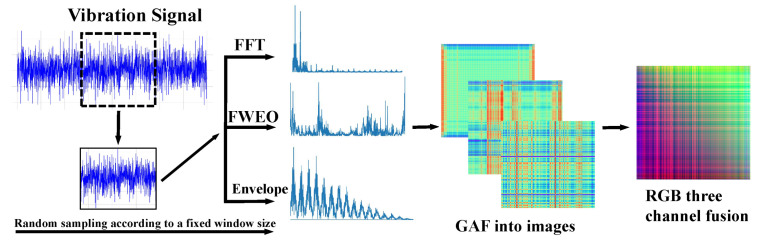
Workflow diagram of FFE.

Figure 2 illustrates the workflow of FFE. FFE employs FFT, FWEO, and Envelope to extract key fault features from the vibration signals, subsequently converting the fused features from the three channels into RGB images using GAF. The experiments presented in Section 5.1 demonstrate that the application of FFE effectively mitigates the adverse effects of variations in working conditions on the accuracy of fault diagnosis.

### 3.3. Lightweight GEFA-Net

**Figure 3:**
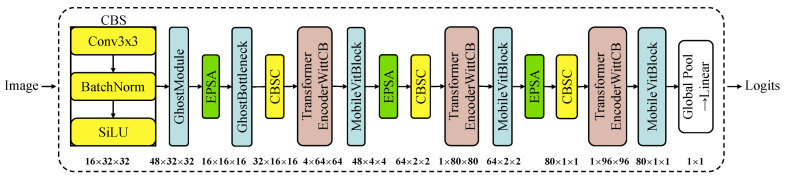
Lightweight GEFA-Net.

The spectral characteristics of a given vibration signal may exhibit variations due to fluctuations in operational parameters such as load, velocity, and lubrication conditions. Such variability necessitates that neural networks possess the capability to intricately capture these evolving feature dynamics. However, conventional neural networks typically demonstrate inadequate adaptability to these changes. Traditional CNN models enhance fault feature extraction by incorporating additional convolutional layers, thereby better adapting to diagnostics under complex operating conditions. This approach not only escalates the risk of overfitting but also increases the number of model parameters, necessitating more computational resources and extended processing time. To address the challenge of adapting traditional models for feature extraction across diverse operational conditions, our advanced deep learning framework, as depicted in Figure 3, integrates local feature extraction with global information synthesis. This framework is designed to significantly enhance accuracy in image classification tasks. The proposed framework markedly improves the model’s capability to discern the inherent complexity of images by amalgamating traditional convolutional mechanisms with enhanced attention strategies and an optimized Transformer encoder. This innovation results in a substantial reduction in the model’s parameter count. Concurrently, we transitioned from the AdamW optimizer to the Lion optimizer, aiming to boost both model performance and training efficiency. The enhancements achieved with the revised GEFA-Net are elucidated in the subsequent sections.

(1) Ghost module, Ghost Bottleneck, and EPSA

**Figure 4:**
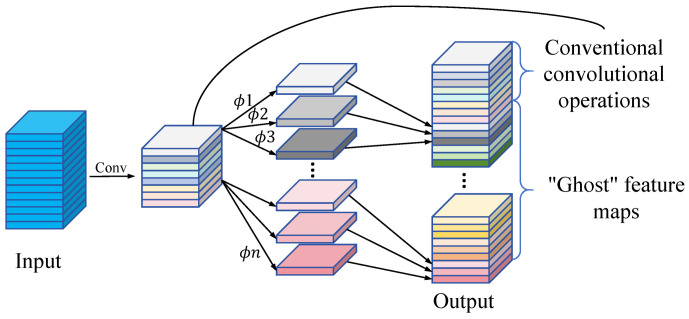
The Ghost module’s linear procedure.

We refine the balance between depth, width, and computational efficiency using the Ghost module, Ghost Bottleneck, and EPSA mechanisms as integrated into the MobileVit model. The Ghost module enhances feature mapping through cost-effective operations, thus reducing computational overhead. As depicted in Figure 4, this module bifurcates the standard convolutional layer into two segments. Initially, conventional convolutional operations are performed and meticulously regulated to minimize the number of convolutions. These operations yield a primary set of feature maps that encapsulate the essential information of the input data. The effectiveness of these enhancements is systematically explored in subsequent analyses. In the subsequent phase of the Ghost module, a linear operation is employed to expand the number of feature maps. This stage leverages the feature maps produced during the initial convolutional process, applying a sequence of economical linear transformations to create additional “ghost” feature maps. These ghost feature maps are designed to effectively encapsulate and amplify the information inherent in the original features. Consequently, the Ghost module facilitates the generation of an augmented set of feature maps, achieving this with reduced parameter count and computational complexity while maintaining the dimensional integrity of the output feature map [54]. The Ghost Bottleneck architecture is composed of two sequentially stacked Ghost modules. The initial Ghost module functions as an expansion layer, amplifying the channel count to enrich the representational capacity of the feature maps. Conversely, the subsequent Ghost module compresses the number of channels to align with the dimensions required by the shortcut paths. These shortcut paths are strategically deployed to integrate the inputs and outputs of both Ghost modules. By executing a series of cost-effective linear operations on each intrinsic feature map, the Ghost Bottleneck effectively multiplies the number of feature maps, thereby enhancing the model’s depth without proportionately escalating the computational burden or parameter count.

We refine the balance between depth, width, and computational efficiency using the Ghost module, Ghost Bottleneck, and EPSA mechanisms as integrated into the MobileVit model. The Ghost module enhances feature mapping through cost-effective operations, thus reducing computational overhead. As depicted in Figure 4, this module bifurcates the standard convolutional layer into two segments. Initially, conventional convolutional operations are performed; these are meticulously regulated to minimize the count of convolutions. Such operations yield a primary set of feature maps, encapsulating essential information of the input data. The effectiveness of these enhancements is systematically explored in subsequent analyses. In the subsequent phase of the Ghost module, a linear operation is employed to expand the number of feature maps. This stage leverages the feature maps produced during the initial convolutional process, applying a sequence of economical linear transformations to create additional “ghost” feature maps. These ghost feature maps are designed to effectively encapsulate and amplify the information inherent in the original features. Consequently, the Ghost module facilitates the generation of an augmented set of feature maps, achieving this with reduced parameter count and computational complexity while maintaining the dimensional integrity of the output feature map [54]. We chose the Ghost module as the core of our approach after extensive theoretical research and empirical comparisons. Our objective was to enhance MobileVit by improving model inference speed, significantly reducing the number of model parameters and simultaneously improving diagnostic accuracy. Among various modules tested, including ALBERT, TinyViT, MiniViT, and DynamicViT, the Ghost module demonstrated superior performance in achieving our lightweighting goals. Specifically, Ghostnet employs fewer parameters to linearly generate new features, which is particularly advantageous for creating efficient lightweight models.

The Ghost Bottleneck architecture is composed of two sequentially stacked Ghost modules. The initial Ghost module functions as an expansion layer, amplifying the channel count to enrich the representational capacity of the feature maps. Conversely, the subsequent Ghost module compresses the number of channels to align with the dimensions required by the shortcut paths. These shortcut paths are strategically deployed to integrate the inputs and outputs of both Ghost modules. By executing a series of cost-effective linear operations on each intrinsic feature map, the Ghost Bottleneck effectively multiplies the number of feature maps, thereby enhancing the model’s depth without proportionately escalating the computational burden or parameter count.

Specifically, within the MobileViTBlock, the conventional convolution operation is enhanced through the integration of the Ghost module. Furthermore, the Ghost module is employed as the initial convolution to enhance the model’s efficiency by facilitating feature extraction at a reduced computational cost during the early stages of the network. Concurrently, the Ghost Bottleneck primarily replaces the bottleneck layers within MobileViT, specifically those following the Inverted Residual layer. This substitution introduces more effective feature processing capabilities to the model, thereby optimizing overall performance. The computational expenditure of these operations is considerably lower compared to standard convolutional operations, thereby significantly enhancing the efficiency of the model. Despite the diminished computational demand and fewer parameters, experimental results demonstrate that the MobileViT model, incorporating both the Ghost module and Ghost Bottleneck structures, exhibits improved recognition performance. This approach further refines the features while preserving the spatial dimensionality of the feature map. Consequently, it circumvents the common issue of parameter proliferation associated with traditional convolutional layers and mitigates the risk of overfitting that often accompanies excessive convolutional layers.

**Figure 5:**
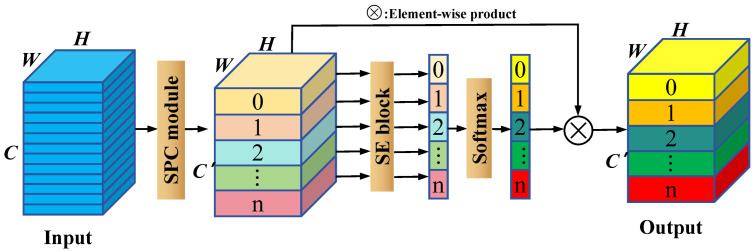
The structure of the EPSA module.

To augment the model’s capacity for discerning and processing critical information, the EPSA has been integrated following the Ghost Bottleneck layer, as detailed in [50]. This integration enhances the model’s representation of global features within the channel dimension by compressing and recalibrating the global content of the feature maps. The deployment process of the EPSA module is systematically outlined in Figure 5, comprising four methodical steps. The Squeeze and Concat (SPC) module first generates a channel-specific multi-scale feature map, consolidating vital data to fortify the foundational analysis. The SE block module then extracts attention vectors from these feature maps at various scales, meticulously emphasizing essential features while discarding non-essential information, thereby improving the model’s computational efficiency. Following this, the vectors are recalibrated using the Softmax function, which aids in deriving channel-specific weights to optimize feature representation and enhance the focus on crucial attributes for achieving the stipulated objectives. This sequence culminates in an element-wise multiplication of the recalibrated weights with the corresponding feature maps, significantly refining the representation’s accuracy and clarity. The resultant feature map, rich in detailed and precise multi-scale feature information, considerably enhances the model’s ability to analyze diverse feature dimensions and elevates its performance and efficiency in complex data analysis scenarios.

Drawing upon Zhang’s investigation [50] and addressing the intricate challenge of bearing fault feature extraction under variable operating conditions, this study deduces that the incorporation of the EPSA module markedly enhances the model’s capability to discern critical features. This is achieved through the adept integration of multi-scale spatial information and a cross-channel attention mechanism. Such enhancements are particularly pronounced in scenarios characterized by complex backgrounds and heterogeneous target scales. Consequently, we strategically assessed the integration of the EPSA module within our model, considering its placement and effectiveness in enhancing feature extraction and representational capacities. Our empirical modifications focused on three critical integration points within the network’s architecture, aiming to optimize the model’s representational and diagnostic accuracy. a. Post-MobileViTBlock Integration: We introduced the EPSA module following each MobileViTBlock, particularly after processing by the self-attention mechanism. This enhancement targets the amplification of multi-scale representational capabilities, which is crucial for capturing detailed intricacies within datasets. b. Pre-Feature Pyramid Network Enhancement: Before linking with the Feature Pyramid Network (FPN), the EPSA module was integrated to strengthen the model’s detection capabilities and augment its segmentation performance. This placement is designed to refine the accuracy with which the model delineates distinct segments in complex images. c. Global Average Pooling Optimization: The placement of the EPSA module prior to the Global Average Pooling (GAP) stage aimed at facilitating the module’s role in the final attention weighting and feature tuning, which is essential for extracting critical features and enhancing diagnostic precision.

Our experimental investigations indicate that the EPSA module’s incorporation post-MobileViTBlock significantly enhances the model’s diagnostic capabilities, especially in analyzing fault signals under variable operational conditions. These conditions require robust multi-scale processing to manage the complexity of feature representations effectively. The results demonstrate the EPSA module’s effectiveness in fortifying the model’s feature representation and underscore its potential in new diagnostic scenarios. This enhancement adaptively responds to diverse operational environments by customizing to specific input features, thus enriching the model’s feature extraction capabilities with greater depth and precision in complex scenarios. Concurrently, this approach substantially reduces computational resource consumption and operational latency. By integrating efficient parameter utilization with a blend of global and local information processing techniques, we have developed a robust framework suitable for complex tasks such as fault diagnosis in intricate settings. This design not only enhances the model’s analytical performance but also streamlines the computational process, facilitating more rapid and accurate diagnostics and classifications.

(2) CB module and CB Integration with Transformer

In the MobileVit framework, the employment of the Transformer architecture significantly enhances the model’s comprehension of intricate image content through the effective capturing of long-range dependencies among image features. This capability is paramount in vision-related tasks as it facilitates the integration of minute local details with expansive global information, thereby refining the overall interpretation of the image’s structure and content. While MobileVit demonstrates exceptional prowess in extracting global information pertinent to fault characteristics, effective troubleshooting under multifaceted operational conditions necessitates a targeted approach to context-specific feature learning. The conventional Transformer architecture, despite its proficiency in managing complex sequential dependencies, does not optimally leverage the global contextual information inherent in high-dimensional image features when applied directly. This limitation can adversely affect the diagnostic capabilities in complex scenarios, thereby diminishing the model’s effectiveness in accurately representing and identifying bearing fault features under challenging operational conditions.

**Figure 6:**
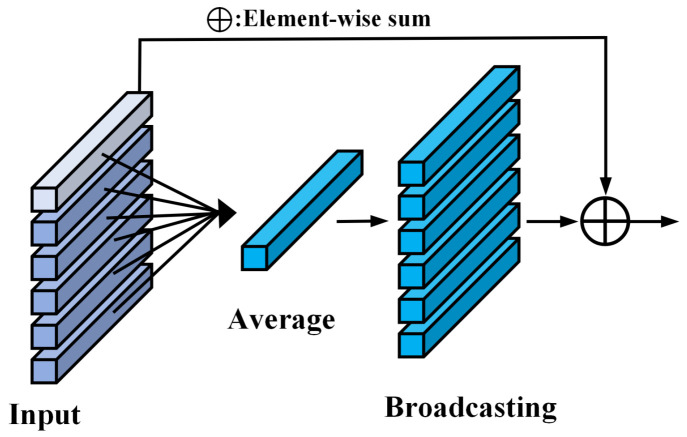
Context broadcasting module.

To address this issue, we have refined the Transformer encoder within the MobileVit architecture by incorporating a CB module [48], as illustrated in Figure 6. This enhancement aims to augment the model’s capability to discern more profound global information pertinent to bearing fault characteristics under diverse operational conditions, thereby elevating diagnostic effectiveness. Specifically, a CB module is integrated into the Transformer to process and amplify global context information, ensuring that the model leverages a synthesized representation of both global and local data at each processing stage. Initially, the CB module computes the mean of the sequence (or feature map) in the spatial dimension to produce a context vector that encapsulates the global context of the entire image. This vector is subsequently redistributed to each position within the sequence and combined with the original features, thereby enriching the local features with global contextual data. The deployment of this mechanism ensures that each token is imbued with a comprehensive representation of global context prior to progressing to subsequent layers, which is vital for an accurate interpretation of both the global structure and intricate details of the image.

**Figure 7:**
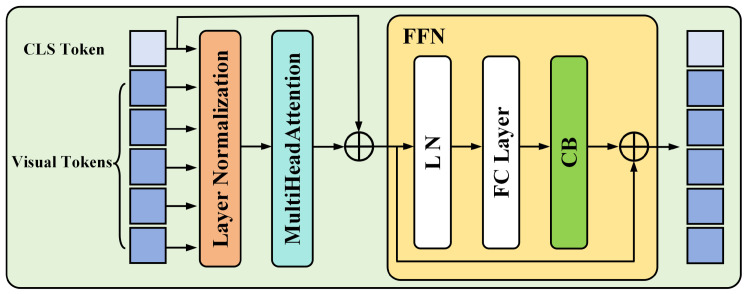
Structure of CB-TransformerEncoder.

We integrate the CB with the Transformer encoder module in the Transformer section, as shown in Figure 7, and the integrated module is named CB-TransformerEncoder. This module is strategically embedded within the feed-forward network (FFN) layer, subsequent to the self-attention mechanism and local information processing. The placement of CB-TransformerEncoder after the FFN is pivotal, as it introduces a global context subsequent to the processing of local features, ensuring that the integration of global context is predicated on a nuanced and enriched representation of local features. Such enhancement significantly fortifies the model’s ability to merge global and local information, thus augmenting the overall completeness and analytical depth of the model. Within the Transformer architecture, while the self-attention layer is primarily responsible for facilitating local-to-local relationship learning, the introduction of the FFN layer adds a layer of nonlinearity, which is crucial for capturing more complex feature representations. The subsequent integration of the CB module encourages the model to further amalgamate this locally processed information with a global context, leveraging its pre-established comprehension of local features and complex non-linear relationships. This strategic arrangement not only optimizes information flow within the model but also substantially enhances its capacity to process and interpret complex data, thereby extending the learning scope and augmenting diagnostic capabilities in intricate scenarios. Furthermore, positioning the CB module prior to or within the self-attention layer could prematurely merge global context with local features, potentially constraining the self-attention mechanism’s capacity to delineate complex relationships among local details. Conversely, situating the CB module subsequent to the FFN layer allows the model to first exhaustively exploit the self-attention mechanism for enhancing the interrelations among local features. The subsequent incorporation of global context through the CB module thus follows, strategically avoiding any premature interference of global information with the local feature delineation process.

The experimental results demonstrate a significant enhancement in the performance of the improved model. Specifically, after 50 training iterations, the diagnostic accuracy exhibits a 4.1% increase relative to the MobileVit model. Moreover, this methodology enables the model not only to discern intricate details among local features but also to integrate global context at each processing stage. Consequently, this enhances the model’s proficiency in identifying bearing fault characteristics under complex operational conditions. This integrated approach of processing both local and global information offers a more holistic and effective strategy for the detailed analysis of image contents.

## 4. Experiment Study and Evaluation Metrics

To validate the effectiveness of the proposed fault diagnosis framework, this study meticulously designs and executes fault diagnosis experiments on rolling bearings under varying operational conditions. Specifically, two datasets are employed for these experiments: the open experimental dataset from bearing failure experiments conducted at the University of Ottawa [55] and a dataset developed by our test rig of experiments. Both datasets are elaborated upon as follows:

### 4.1. Bearing Fault Test Rig and Dataset of University of Ottawa

#### 4.1.1. Introduction of Test Rig

**Figure 8:**
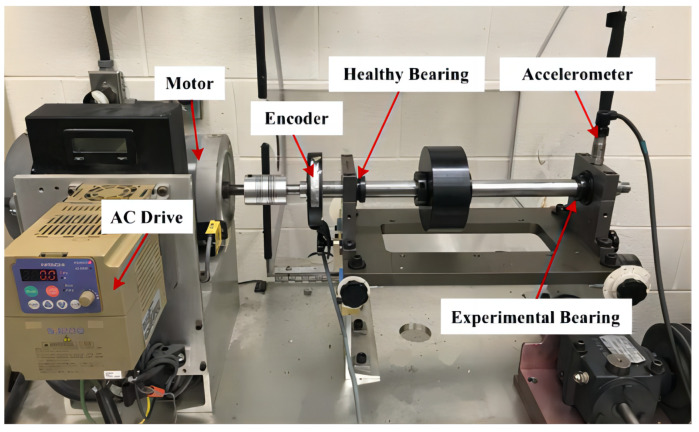
Test rig of Ottawa.

The Department of Mechanical Engineering at the University of Ottawa employed the SpectraQuest’s Mechanical Failure Simulator (MFS-PK5M) for the acquisition of bearing vibration data. The experimental setup, depicted in Figure 8 [56], comprises a motor-driven shaft, the speed of which is regulated by an AC drive. This shaft is supported by two ER16K ball bearings; a healthy bearing is installed on the left side, while the right side houses an experimental bearing that is replaceable to accommodate various health conditions. Vibration data were collected using an accelerometer (ICP Accelerometer Model 623C01) mounted on the housing of the experimental bearing. Additionally, an incremental encoder (EPC model 775) was utilized to monitor the shaft speed.

#### 4.1.2. Introduction of Experimental Datasets

The University of Ottawa dataset comprises vibration signals collected from bearings subjected to various health and speed conditions. This dataset categorizes the bearings into three health conditions: healthy, inner ring failure, and outer ring failure. Additionally, the operational speeds were varied across four conditions: increasing speed, decreasing speed, increasing followed by decreasing, and decreasing followed by increasing. For each experimental configuration, three trials were performed, resulting in a total of 36 datasets. The data acquisition was executed at a sampling rate of 200,000 Hz over a duration of 10 s per sample. A detailed description of the datasets is provided in Table 2.

### 4.2. Our Test Rig and Dataset

#### 4.2.1. Introduction of Our Test Rig

**Figure 9:**
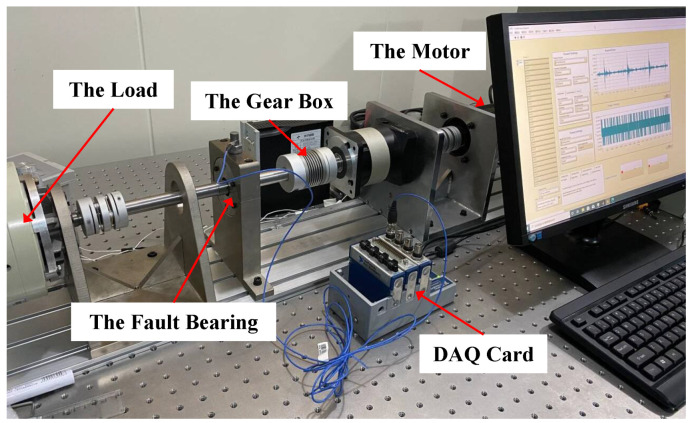
Our test rig.

To further validate the effectiveness and advantages of the GEFA-Net, we conducted fault diagnosis experiments under varying operational conditions using our specialized test rig. This rig, illustrated in Figure 9, comprises an AC servo motor, a magnetic particle brake, a planetary gearbox with a reduction ratio of 3, and a test bearing (model: SKF6204). The AC servo motor’s operations are regulated by a servo controller, while the magnetic particle brake induces load. Vibration data from the rolling bearing were collected under diverse loads and speeds using miniature accelerometers (Model No. PCB352C03) affixed to the test bench. These signals were captured via a DAQ card (Model: NI 9234), with a consistent sampling frequency set at 2.56 kHz.

#### 4.2.2. Introduction of Our Experimental Datasets

**Figure 10:**
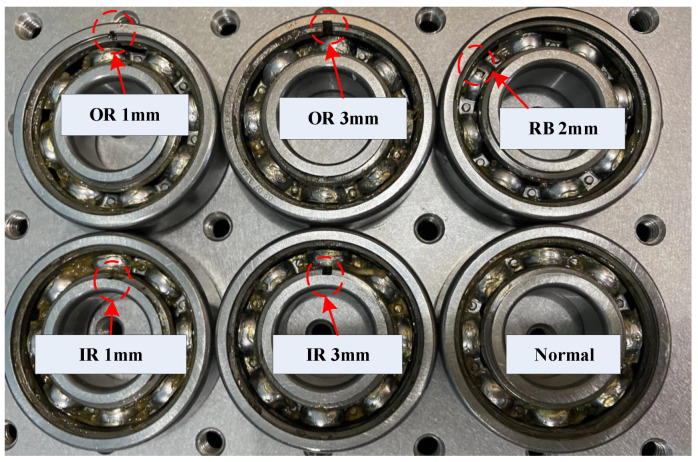
Our experimental datasets.

Our datasets comprise bearings classified into four health conditions: Normal, Inner Ring Fault, Outer Ring Fault, and Roller Bearing Fault. Specifically, as shown in Figure 10, Outer Ring Faults are categorized into two groups with fault widths of 1 mm (OR 1 mm) and 3 mm (OR 3 mm), while Inner Ring Faults are similarly divided into two groups with fault widths of 1 mm (IR 1 mm) and 3 mm (IR 3 mm). Roller Bearing Faults are characterized by a single fault width of 2 mm (RB 2 mm). To comprehensively simulate complex working conditions, the operating speed varies across six different settings. Experimental configurations for each condition are replicated across five trials, resulting in a total of 6 datasets encompassing 36 variable fault scenarios. Each fault condition is sampled with 400,000 data points, yielding datasets comprising 5000 samples each.

### 4.3. Experimental Settings

The experiments are executed on a computing setup featuring an AMD Ryzen 7 5800H with Radeon Graphics (3.2 GHz, 16 GB RAM) and an NVIDIA GeForce RTX 3060 Laptop GPU (CUDA version 12.1), utilizing Python 3.9.12 with PyTorch 2.1.2. These experiments employ the Lion optimizer, initiated with a learning rate of 0.0001. The batch size and number of epochs are set at 50 each. A total of 5000 fault samples from both the Ottawa dataset and our dataset for the confiscated class are utilized. Each dataset is randomly divided into training, validation, and test sets in a 6:2:2 ratio [57]. Dr. Andrew Ng, an Associate Professor in both the Department of Computer Science and the Department of Electrical Engineering at Stanford University, endorses this split as it offers a balanced approach to mitigating overfitting while ensuring sufficient evaluation of the model’s performance on unseen data.

### 4.4. Evaluation Metrics

To comprehensively assess the performance and merits of the proposed model, we utilize key evaluation metrics, including accuracy, precision, recall, false alarm rate, miss rate, detection speed, and the number of floating-point operations per second (FLOPs) [58]. We illustrate the model’s reliability in practical scenarios by depicting a confusion matrix, which vividly demonstrates the model’s capabilities in identifying various fault types across the metrics of accuracy, precision, recall, false alarm rate, and miss rate. Comparative experiments with alternative network models quantitatively showcase the superior performance of our enhanced model. This evaluation includes comparisons of parameter counts, detection speeds, and accuracies, thereby underscoring the model’s significance in the domain of rolling bearing fault diagnosis. Additionally, we evaluate the model’s inference time, aggregating and averaging the inference times across all test set data to determine the model’s average inference time. Finally, the effectiveness of each innovation within the improved network model is validated through ablation studies, thereby reinforcing the necessity and impact of the proposed enhancements.

## 5. Experimental Results

### 5.1. Comparative Experiment

#### 5.1.1. Results and Discussion Based on the Data Experiment of the University of Ottawa

(1) Effectiveness of FFE

Section 4.1.2 introduces the University of Ottawa Bearing Failure Experiment dataset, noting that all data are sampled at a rate of 200,000 Hz with a duration of 10 s per sample, resulting in each dataset containing over 2,000,000 data points. Consequently, we establish a sampling window of 2000 and randomly select 200 windows per dataset to enhance sample diversity. The FFE employs several transformations: initially, it computes the signal using FFT; then, it applies envelope detection and an FWEO. Subsequently, the data are transformed into images through a Gram angle field technique, followed by the fusion of the three channels.

**Figure 11:**
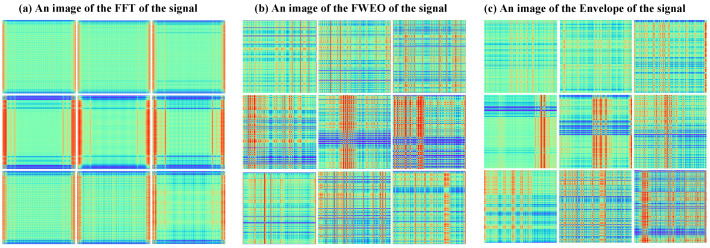
RGB image after three-channel feature extraction. (**a**) presents the images transformed via FFT, (**b**) displays images transformed by the FWEO, and (**c**) shows the images processed through envelope detection.

Following the FFE’s feature extraction, the transformed signals are represented as 2D image signals, depicted in Figure 11. For illustration, datasets H-B-1, I-B-1, and O-B-1 are selected. Figure 11a displays the images transformed via FFT, Figure 11b shows images transformed by the FWEO, and Figure 11c presents the images processed through envelope detection. The first row illustrates the transformation results of the healthy signal, the second row represents the Inner Ring Fault signal, and the third row visualizes the Outer Ring Fault signal. Each row comprises three images corresponding to the aforementioned transformations.

Figure 11a illustrates that both inner and outer fault signals display distinct red and blue linear structures, indicative of signal periodicity. In contrast, healthy signals manifest lower vibrational components and subdued periodic patterns, with noise potentially obscuring these characteristics and complicating the differentiation from fault signals. Consequently, the transformed image of the healthy signal appears more diffuse yet retains the linear structure characteristic of periodicity. Figure 11b captures the frequency characteristics of each signal type, particularly highlighting the fault vibration frequencies associated with the inner and outer rings of the bearing through color variations. Figure 11c distinctly differentiates fault signals, showing that the rate of change in the Inner Ring Fault signal surpasses that of the outer ring, and the fault frequency of the inner ring is higher than that of the outer. In the outer-ring fault signal, the red and blue linear configurations exhibit a higher degree of order, signifying clear periodic stability. Noise significantly influences the healthy signal, resulting in a more dispersed image representation. Collectively, these transformations underscore the effectiveness of the feature extraction process, enhancing the visibility of critical fault signals and accentuating the distinctions among the various signal types, thereby demonstrating the success of critical feature extraction.

(2) FFE comparison experiment

**Figure 12:**
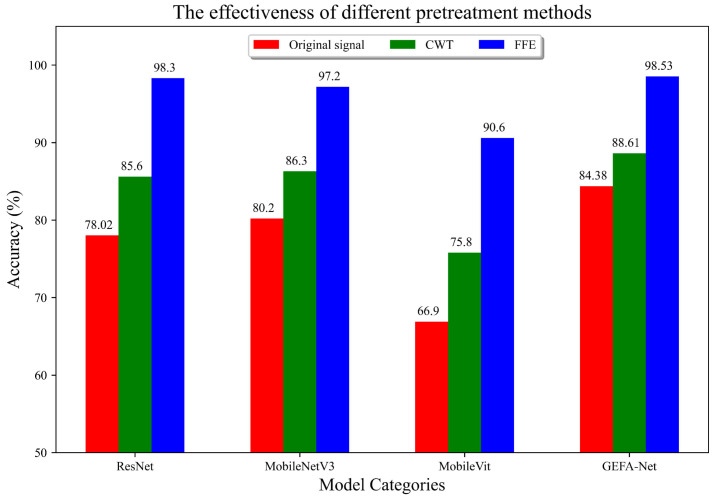
Experimental comparison of three preprocessing methods (Original signal, CWT, FFE) across different models.

In this section, we assess the performance of the proposed preprocessing FFE, comparing it against both the original signal and signals processed using Continuous Wavelet Transform (CWT). To demonstrate the superiority of FFE, we convert the original signal and the CWT-processed signal into 2D images using the GAF technique. These images, along with those processed by FFE, are then subjected to training using various neural network architectures, including ResNet, MobileNetV3, MobileViT, and GEFA-Net, with the number of epochs set at 50. The diagnostic outcomes from this comparative study further corroborate the effectiveness of the FFE. The experimental results are depicted in Figure 12.

The experimental findings demonstrate that the MobileViT model achieves the most substantial performance enhancement, with an improvement of 23.7% in accuracy following the application of FFE, compared to direct training on the original signal. Similarly, ResNet exhibits a 20.28% increase in accuracy, while MobileNetV3 and GEFA-Net show improvements of 17% and 14.15%, respectively. Notably, all four models significantly outperform those trained with CWT preprocessing in terms of diagnostic accuracy. These comparative results affirm the effectiveness of the FFE in extracting critical fault features under variable operating conditions.

(3) Model comparison experiment

To conduct a thorough comparative analysis of the models proposed in this study, we evaluated each model based on inference speed, test accuracy, FLOPs, parameter count, and model size. Among these, inference speed is regarded as the most crucial measure for lightweighting, and we use the average inference time to test a single piece of data as our speed metric. The significance of inference speed is underscored by its direct impact on the real-time applicability of models in practical scenarios. By prioritizing models with lower inference times, we ensure that the deployed solutions are not only efficient but also responsive under operational conditions. This approach facilitates the identification of models that strike an optimal balance between computational efficiency and diagnostic accuracy, thereby enhancing their suitability for implementation in resource-constrained environments. Our comprehensive evaluation framework allows for a nuanced understanding of each model’s performance across multiple dimensions, ensuring a robust and holistic assessment of their capabilities. For comparison, we chose well-established models, such as MobileViT, MobileNetV3, Swin Transformer, Vision Transformer, and ResNet, following established research protocols. To underscore the significant reduction in model parameters achieved by GEFA-Net, we consistently selected the configurations with minimal parameter counts across all models. Specifically, MobileViT was evaluated using the mobile_vit_small configuration, MobileNetV3 with mobilenet_v3_small, Swin Transformer with Swin_base_patch4_window7_224, Vision Transformer with vit_base_patch16_224_in21k, and ResNet with resnet34. This selection criterion facilitates an equitable comparison and highlights the efficiency of our approach to parameter reduction. Initially, the models were trained and evaluated using the dataset outlined in Section 4.3, from which we derived training loss and test accuracy for each configuration. The subsequent calculations of FLOPs, parameters, and model sizes were performed using the THOP library within the PyTorch environment. As detailed in Table 3, MobileVit, with an input size of 3×64×64, registers 21.73 million FLOPs, a model size of 3.83 MB, and 1.15 million parameters. In contrast, GEFA-Net achieves considerably lower FLOPs at 7.88 million, with a model size of 3.93 MB and 0.96 million parameters, thereby indicating a substantial enhancement in computational efficiency. While MobileNetV3 and ResNet demonstrate commendable accuracy, their computational demands are significantly higher, with FLOPs of 22.81 million and 300.78 million, respectively, and larger model sizes and parameter counts than GEFA-Net. These comparisons highlight GEFA-Net’s distinct advantages in terms of computational cost and efficiency while maintaining competitive accuracy levels. The Swin Transformer and Vision Transformer exhibit lower accuracy compared to other models, and they require substantially higher computational resources, with FLOPs reaching 740.28 million and 1472.00 million, respectively. Additionally, their substantial model sizes of 108.24 MB and 393.19 MB, along with parameter counts of 28.27 million and 103.03 million respectively, underscore their limitations in resource-constrained environments. To assess diagnostic speed, we calculated the average inference time needed for each model to process the data once. MobileVit recorded an inference time of 13.32 ms, while GEFA-Net achieved a faster time of 9.78 ms, closely approaching MobileNetV3’s 9.27 ms. ResNet shows a slightly lower inference time compared to MobileVit. In contrast, the Vision Transformer and Swin Transformer have longer inference times, at 24.42 ms and 28.93 ms, respectively. In conclusion, GEFA-Net not only attains a high level of accuracy but also demonstrates significant efficiency regarding FLOPs, model size, and parameter count. This efficiency underscores the model’s advantageous balance between high accuracy and low computational demand, rendering GEFA-Net particularly suitable for use in resource-constrained environments.

**Figure 13:**
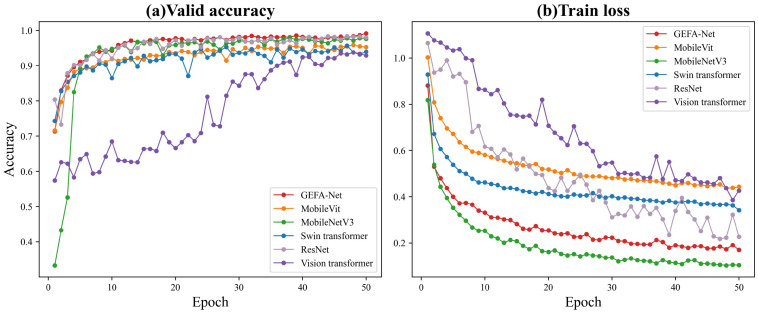
Comparison of model accuracy with Ottawa datasets. (**a**) Valid accuracy for 50 training epochs per model. (**b**) Train loss for 50 training epochs per model.

Table 4 and Figure 13 illustrate the accuracy and error metrics of each model on the test set throughout the training period. Notably, the training accuracy of model GEFA-Net attains 98.53% with an input size of 3×64×64. In contrast, the original MobileVit model manifests a markedly lower accuracy of 94.4% under identical conditions, underscoring a significant enhancement of 4.1 percentage points in our model. Upon reaching training stability, MobileNetV3 demonstrates an accuracy of 97.2%, whereas ResNet experiences initial fluctuations but subsequently stabilizes at an accuracy of 98.2%. Both the Swin Transformer and Vision Transformer, representing classical Transformer architectures, exhibit slightly inferior performance post-stabilization, achieving accuracies of 95.21% and 94.27%, respectively. Comparative experimental outcomes affirm that all models exhibit lower testing accuracies compared to our enhanced model GEFA-Net. Moreover, GEFA-Net showcases pronounced convergence characteristics, achieving stability by the 10th epoch and recording a minimum loss of 0.17, which is substantially lower than MobileVit’s minimum loss of 0.44. Except for MobileNetV3, which records a minimum loss of 0.11, all other models present higher training losses than GEFA-Net. These findings corroborate the superior effectiveness of GEFA-Net in parameter training and loss optimization, significantly enhancing its learning capabilities.

**Figure 14:**
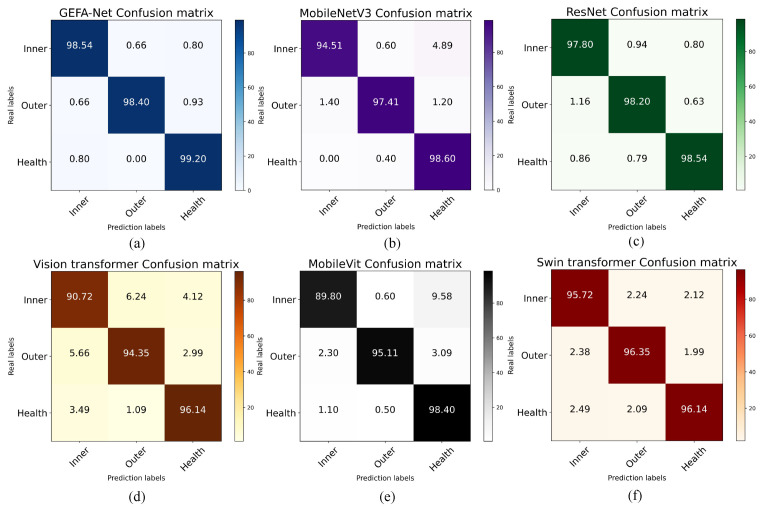
Confusion matrix for the Ottawa datasets. (**a**) presents the GEFA-Net Confusion matrix, (**b**) displays the MobileNetV3 Confusion matrix, (**c**) shows the ResNet Confusion matrix, (**d**) presents the Vision transformer Confusion matrix, (**e**) displays the MobileVit Confusion matrix, (**f**) shows the Swin transformer Confusion matrix.

The confusion matrix serves as an essential tool for evaluating the performance of classification models, enabling the visualization of the correlation between predicted outcomes and true labels. This matrix facilitates the derivation of critical performance metrics, including accuracy, precision, recall, false alarm rate, and miss rate. As depicted in Figure 14, the confusion matrix for the model under discussion is presented. Notably, GEFA-Net demonstrates substantial advantages in managing intra-class and inter-class errors. Specifically, it excels in identifying inter-class discrepancies, achieving the highest accuracy relative to competing models. The model distinctly surpasses other models in identifying within-class errors, offering a significant advantage in the fault diagnosis of bearings under complex operational conditions. Specifically, MobileNetV3 exhibits robust classification capabilities for the first and second categories, yet it demonstrates a pronounced misclassification rate within the first category, frequently misclassifying samples from the first into the third category. In contrast, ResNet maintains a balanced performance across all categories, characterized by high accuracy and a minimal rate of misclassification. Although the Swin Transformer achieves relatively even performance across the three categories, it experiences a slight increase in the misclassification rate for each category, likely attributable to the architecture’s increased sensitivity to inter-category similarity in complex image categorization tasks. Conversely, MobileVit’s performance under complex working conditions remains suboptimal, with a particularly notable misclassification rate in the first category of tasks. The Vision Transformer is the least effective, possibly due to inadequate local feature capture and insufficient feature extraction capacity under variable operational conditions. Overall, GEFA-Net emerges as the superior model in this comparative analysis, particularly excelling in classification accuracy and model efficiency.

#### 5.1.2. Based on the Results and Discussion of Our Bearing Fault Data Experiment

(1) FFE comparison experiment

**Figure 15:**
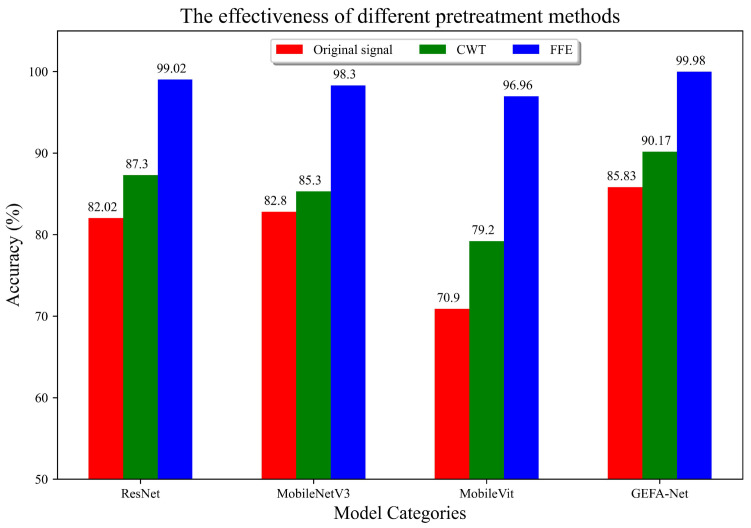
Experimental comparison of three preprocessing methods (Original signal, CWT, FFE) using our dataset across different models.

The experimental findings depicted in Figure 15 demonstrate that the fault-critical features extracted by FFE are markedly superior to those derived from the original signal and Continuous CWT. Specifically, the MobileVit model exhibits the most substantial enhancement, with an improvement of 26.96%, followed by ResNet at 17%, MobileNetV3 at 15.5%, and GEFA-Net at 14.15%. Notably, the diagnostic accuracies of all four models exceed those achieved with the CWT following the application of FFE. This substantiates the effectiveness of FFE in isolating pivotal fault characteristics under various operational conditions.

(2) Model comparison experiment

**Figure 16:**
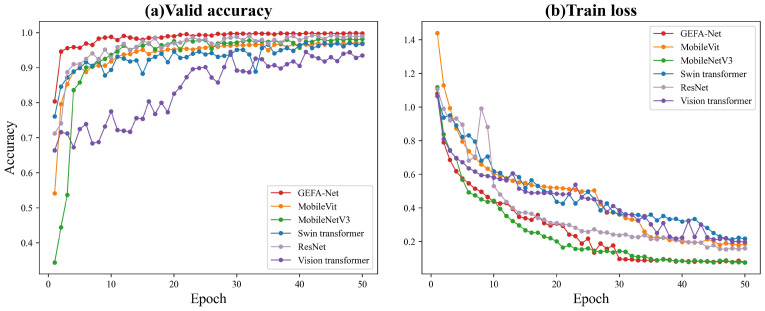
Comparison of model accuracy with our datasets. (**a**) Valid accuracy for 50 training epochs per model. (**b**) Train loss for 50 training epochs per model.

Table 5 and Figure 16 present the accuracy and error metrics of each model on the test set during training. An analysis of the training loss and validation accuracy dynamics reveals that the GEFA-Net model demonstrates a profound reduction in loss from an initial 1.18 to 0.077 throughout the training sequence, accompanied by an increase in validation accuracy from 0.804 to 0.999. These marked improvements underscore our model’s superior loss optimization and learning effectiveness. In contrast, while competing models also show reductions in loss and enhancements in accuracy, GEFA-Net consistently achieves lower loss levels and higher accuracies. Notably, in the later stages of training, GEFA-Net displays a more pronounced accuracy advantage compared to ResNet and Vision Transformer. The validation accuracy of GEFA-Net reaches 99.98% upon training stabilization. Meanwhile, under identical conditions, the original MobileVit model records an accuracy of only 96.9%. Post-stabilization, MobileNetV3 reports an accuracy of 98.3%, while ResNet displays initial fluctuations but eventually achieves 99.02%. Both the Swin Transformer and the Vision Transformer exhibit slightly inferior performance post-stabilization, with accuracies of 97.15% and 95.54%, respectively. The comparative analysis indicates that all models test at lower accuracies than our proposed GEFA-Net model. In summary, GEFA-Net demonstrates exemplary performance on this task, with enhanced learning and generalization capabilities relative to alternative models.

**Figure 17:**
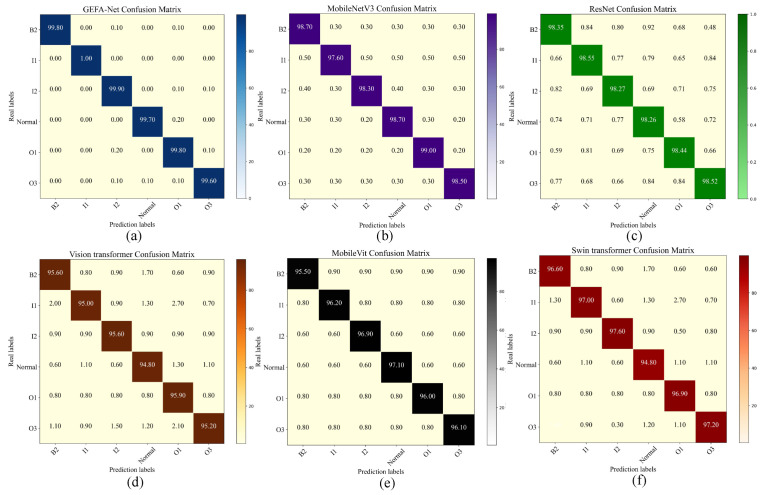
Confusion matrix for our datasets. (**a**) presents the GEFA-Net Confusion matrix, (**b**) displays the MobileNetV3 Confusion matrix, (**c**) shows the ResNet Confusion matrix, (**d**) presents the Vision transformer Confusion matrix, (**e**) displays the MobileVit Confusion matrix, (**f**) shows the Swin transformer Confusion matrix.

Figure 17 displays the confusion matrix derived from the evaluations conducted on our dataset. The matrix reveals that our model, GEFA-Net, exhibits enhanced accuracy across all categories, markedly outperforming competing architectures such as MobileNetV3, ResNet, Swin Transformer, Vision Transformer, and MobileVit in the identification of inter-class discrepancies. Specifically, MobileNetV3 demonstrates deficiencies in discriminating between the third and fourth categories. ResNet, while maintaining a balanced performance across the six categories, registers marginally lower metrics overall. The Swin Transformer achieves a more uniform performance across three categories, yet it incurs a slightly elevated misclassification rate in each category compared to other models. Vision Transformer maintains consistent performance across all categories but exhibits marginally reduced accuracy relative to other high-performing models. Overall, GEFA-Net emerges as the superior model in this comparative analysis, particularly excelling in classification accuracy, a critical factor in the task of diagnosing bearing faults in complex environments.

### 5.2. Ablation Experiment

**Figure 18:**
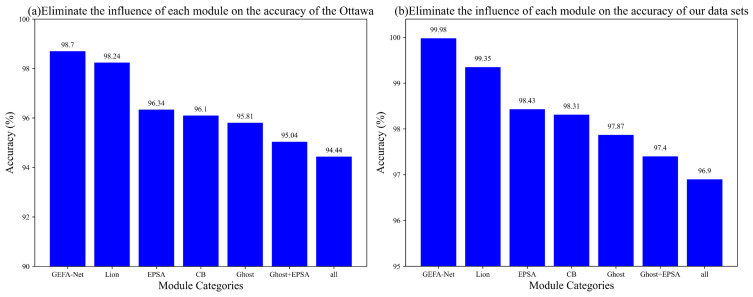
Comparison results of ablation experimental models. (**a**) Ottawa datasets. (**b**) Our datasets.

In this section, we assess the individual contributions of each component within the enhanced network model via a series of ablation studies. Figure 18a,b depict the diagnostic accuracies achieved by our model, GEFA-Net, under various configurations on the Ottawa dataset and our dataset, respectively. These configurations include the substitution of the Lion optimizer, the omission of the EPSA module, the isolated ablation of the CB module, the removal of the Ghost module, the elimination of the combined EPSA and Ghost modules, and the removal of all aforementioned modules. The results indicate that the EPSA, CB, and Ghost modules substantially bolster the model’s performance, with their exclusion leading to significant declines in accuracy. Notably, the integration of the EPSA and Ghost modules exerts the most profound impact on the network’s effectiveness.

## 6. Conclusions

This study introduces a new approach to bearing fault diagnosis under variable working conditions, addressing the complexities of neural networks and the computational demands of existing methodologies. Our proposed framework, the Lightweight Ghost Enhanced Feature Attention Network, integrates the FFE tri-channel preprocessing module with innovative attention-based mechanisms to achieve robust and efficient fault detection across diverse operational conditions. Simultaneously, the attention mechanism within GEFA-Net, implemented through the Ghost Module and EPSA, enhances feature representation and reduces computational demands by generating additional feature maps through linear operations. Our framework achieves significant advancements, with a 63.74% reduction in parameter count compared to MobileVit, while maintaining high diagnostic accuracies of 98.53% and 99.98% for their respective datasets. These results underscore the efficacy of our approach in balancing computational efficiency with diagnostic precision. Empirical evaluations, conducted using datasets from the University of Ottawa and our experimental setup, consistently validate the framework’s proficiency in accurately diagnosing complex machinery faults with attention-based mechanisms and lightweight model architectures. These results underscore the suitability of our framework for real-world industrial applications. Our proposed solution offers a promising approach for bearing fault diagnosis across diverse operational conditions.

## Figures and Tables

**Table 1:** Steps in the work of the framework.

Steps	Descriptions
Step 1	Vibration signals are inputted into the FFE. Subsequently, FFE executes random intercepts based on predetermined window sizes and quantities.
Step 2	FFE conducts three-channel feature extraction for each window. Then, FFE generates a 2D image from the feature-extracted 1D signal using GAF and fuses the three channels into a single RGB image.
Step 3	The FFE splits the generated RGB images into a training set, a validation set, and a test set, in a random manner, following a 6:2:2 ratio.
Step 4	Input the dataset into GEFA-Net for the purpose of diagnosis.

**Table 2:** Experimental datasets of Ottawa.

Bearing Health Conditions	Increasing Speed	Decreasing Speed	Increasing Then Decreasing Speed	Decreasing Then Increasing Speed
Healthy	H-A-1	H-B-1	H-C-1	H-D-1
	H-A-2	H-B-2	H-C-2	H-D-2
	H-A-3	H-B-3	H-C-3	H-D-3
Inner race fault	I-A-1	I-B-1	I-C-1	I-D-1
	I-A-2	I-B-2	I-C-2	I-D-2
	I-A-3	I-B-3	I-C-3	I-D-3
Outer race fault	O-A-1	O-B-1	O-C-1	O-D-1
	O-A-2	O-B-2	O-C-2	O-D-2
	O-A-3	O-B-3	O-C-3	O-D-3

**Table 3:** Comparison of model performance.

Model	Input Size	FLOPs	Model Size	Params	Time
MobileVit	3×64×64	21.73 M	3.83 MB	1.15 M	13.32 ms
MobileNetV3	3×64×64	22.81 M	22.18 MB	5.48 M	9.27 ms
ResNet	3×64×64	300.78 M	83.30 MB	21.80 M	12.5 ms
Swin Transformer	3×64×64	740.28 M	108.24 MB	28.27 M	28.93 ms
Vision Transformer	3×64×64	1472.00 M	393.19 MB	103.03 M	24.42 ms
GEFA-Net	3×64×64	7.88 M	3.93 MB	0.96 M	9.78 ms

**Table 4:** Comparison of model accuracy.

Model	MobileVit	MobileNetV3	ResNet	Swin Transformer	Vision Transformer	GEFA-Net
Accuracy	94.44%	97.2%	98.2%	95.21%	94.27%	98.53%

**Table 5:** Comparison of model performance.

Model	MobileVit	MobileNetV3	ResNet	Swin Transformer	Vision Transformer	GEFA-Net
Accuracy	96.9%	98.3%	99.02%	97.15%	95.54%	99.98%

## Data Availability

The original contributions presented in the study are included in the article.

## Abbreviations

The following abbreviations are used in this manuscript:

GEFA-Net	Ghost Enhanced Feature Attention Network
FFT	Fast Fourier Transform
FWEO	Frequency Weighted Energy Operator
EPSA	Efficient Pyramid Squared Attention
EMD	Empirical Mode Decomposition
SVD	Singular Value Decomposition
WT	Wavelet Transform
WPT	Wavelet Packet Transform
EWT	Empirical Wavelet Transform
VMD	Variational Mode Decomposition
PCA	Principal Component Analysis
SVM	Support Vector Machines
ANN	Artificial Neural Networks
ResNet	Residual Networks
GAF	Gramian Angular Field
ECA	Efficient Channel Attention
MTF	Markov Transfer Fields
ViT	Vision Transformer
SiT	Signal Transformer Neural Network
AM	Amplitude Modulation
TFT	time–frequency Transformer
TCN	Transformer Convolution Network
CB	Context Broadcasting
CNN	Convolutional Neural Network
SPC	Squeeze and Concat
FPN	Feature Pyramid Network
GAP	Global Average Pooling
FFN	Feed-forward network
FLOPs	floating-point operations per second
CWT	Continuous Wavelet Transform
